# Signal Analysis and Waveform Reconstruction of Shock Waves Generated by Underwater Electrical Wire Explosions with Piezoelectric Pressure Probes

**DOI:** 10.3390/s16040573

**Published:** 2016-04-22

**Authors:** Haibin Zhou, Yongmin Zhang, Ruoyu Han, Yan Jing, Jiawei Wu, Qiaojue Liu, Weidong Ding, Aici Qiu

**Affiliations:** State Key Laboratory of Electrical Insulation and Power Equipment, Xi’an Jiaotong University, Xi’an 710049, China; zhouhaibin@stu.xjtu.edu.cn (H.Z.); hpeb2006@126.com (Y.Z.); han.ruoyu@stu.xjtu.edu.cn (R.H.); jingyan@stu.xjtu.edu.cn (Y.J.); wjw.55.66@stu.xjtu.edu.cn (J.W.); ellery3136677@stu.xjtu.edu.cn (Q.L.); qiuac@mail.xjtu.edu.cn (A.Q.)

**Keywords:** shock wave measurement, pressure waveform reconstruction, PCB138 series pressure probe, Müller-plate needle hydrophone, incident angle, time of flight (TOF) method, FFT

## Abstract

Underwater shock waves (SWs) generated by underwater electrical wire explosions (UEWEs) have been widely studied and applied. Precise measurement of this kind of SWs is important, but very difficult to accomplish due to their high peak pressure, steep rising edge and very short pulse width (on the order of tens of μs). This paper aims to analyze the signals obtained by two kinds of commercial piezoelectric pressure probes, and reconstruct the correct pressure waveform from the distorted one measured by the pressure probes. It is found that both PCB138 and Müller-plate probes can be used to measure the relative SW pressure value because of their good uniformities and linearities, but none of them can obtain precise SW waveforms. In order to approach to the real SW signal better, we propose a new multi-exponential pressure waveform model, which has considered the faster pressure decay at the early stage and the slower pressure decay in longer times. Based on this model and the energy conservation law, the pressure waveform obtained by the PCB138 probe has been reconstructed, and the reconstruction accuracy has been verified by the signals obtained by the Müller-plate probe. Reconstruction results show that the measured SW peak pressures are smaller than the real signal. The waveform reconstruction method is both reasonable and reliable.

## 1. Introduction

Underwater shock waves (SWs) have been widely applied in extracorporeal lithotripsy [1], electrohydraulic forming [2], food non-thermal processing [3,4], SW reservoir stimulation [5,6] and many other fields. The precise measurement of the temporal pressure profiles of underwater SWs is essential in studying the SW characteristics, including peak pressure, energy, impulse and frequency domain characteristics. Furthermore, it is very helpful in estimating explosion power and the possible shock damage caused by SWs.

Various methods have been used to measure SW pressure under different conditions. These measurement methods can be divided into direct and indirect ways. If the measured physical quantity is pressure, the corresponding method is called here a direct method. Otherwise, the method is called an indirect method.

Among the indirect methods, shadowgraph and Schlieren methods [7] can obtain the SW boundary speed based on the time of flight (TOF) method, and then the peak pressure can be calculated with the help of the equation of state (EOS) of water [8]. The laser interference methods [9] can give the spatial distribution of density of water, and then the SW pressure can also be obtained. However, the time-dependent pressure properties are unavailable with the three above methods, so the SW energy cannot be calculated. These optical methods are mainly used to measure high pressures near the blast source. Another good choice for underwater high-pressure measurement is the fiber optic probe hydrophone. This kind of indirect pressure sensor detects the refractive index of water at the fiber-water interface, which varies with the water density [10]. The main disadvantage of this method is the relatively lower sensitivity (for example 2 mV/MPa for a FOPH 2000 device made by RP Acoustics, Lautenbach, Germany), so it is more suitable for high-pressure measurements. Ruby crystals are also adopted as indirect pressure sensors due to their R-line shift effect under pressure [11]. Generally, these indirect diagnostic methods are efficient and precise enough to measure high pressures (several GPa), but the complexity of devices, constraints of the application conditions and the high cost severely limits their practical applications, especially outside the laboratory.

The direct methods for diagnosing SWs are usually based on pressure probes. These probes can be divided into four main categories, *i.e.*, the piezoresistive, the piezoelectric, the capacitive and the inductive type. The piezoresistive and piezoelectric types are now widely studied and used. The manganese-copper manometer is a kind of piezoresistive pressure probe, usually used to measure ultrahigh pressures, which can be as high as ~100 GPa, but it has the shortcoming of large deviations and is hard to work with under discharge conditions [12]. Carbon-based pressure sensors, as another piezoresistive sensor type, have higher sensitivity, but bad linearity, and are often used to measure the arrival time of SWs [13,14]. Quartz, piezoelectric ceramics, tourmaline, polyvinylidene fluoride (PVDF), *etc.* are all common materials used to produce piezoelectric pressure probes. Quartz probes are not hydrostatically sensitive, and their edges must be constrained, which may reduce the resonant frequency and decrease the available bandwidth [15]. Besides, this kind of probe has to be well protected from the SW, because it is easily destroyed by high pressure [13] if placed near the blast source. Tourmaline, as a kind of natural crystal, is hydrostatically sensitive, and has higher piezoelectric constants. PVDF, as a soft and easily shaped material, has been well developed in recent decades. PVDF has a fast rise time and large measurement range, and is very suitable for the pressure measurement of blast shock waves [16,17]. These pressure probe-based measuring methods can obtain the temporal pressure waveform directly, so the SW impulse, energy and other parameters can be calculated easily. Besides, the easy installation and replacement of pressure probes make them more practical and important in real applications. Therefore, it is very meaningful and necessary to investigate SWs by adopting the probe-based pressure sensors.

However, it is very difficult to measure SW precisely with pressure probes, because the SW signal has a large peak pressure and a nanosecond time scale rise time [18]. Besides, SWs generated by UEWEs have much shorter pulse widths (tens of μs) than SWs generated by traditional chemical explosions, which have a pulse width of hundreds to thousands of milliseconds. This will require a much higher frequency response. The precise measurement of SWs generated by UEWEs with pressure probes has to overcome a series of difficulties as follows:

- The SW peak pressure can reach several to tens of GPa near the blast source [8,19,20,21] and tens to hundreds of MPa in the rather far field [22], so a reliable mechanical strength and proper structure are required to avert possible damage to the pressure sensors. Besides, the pressure sensors often work totally immersed in water, which calls for an impeccable waterproofing treatment.
- A proper acoustic impedance matching and effective electromagnetic shielding are also necessary to reduce the signal distortion and noise [13,21].
- This ultra-wideband SW signal puts very high demands on the frequency response characteristics of pressure sensors. In order to ensure a large enough sensitivity, the sensitive element of a pressure sensor cannot be designed too small, but a finite size will stretch the wavefront due to the integrating effect. Besides, the pressure probe is often placed in water at a certain depth and inevitably connected with signal conditioning and recording devices with a rather long cable, then the long cable and the signal conditioning device will greatly distort the waveform and decrease the bandwidth.

Because of the above reasons, the pressure waveforms obtained by pressure probes are often heavily distorted, so restoring the correct pressure waveform from the anamorphic one is very important. In this paper, we selected two reliable, widely used and commonly accepted pressure probes, *i.e.*, the tourmaline-based PCB138 series pressure probe and the PVDF-based Müller-plate needle hydrophone, as our study objects. Firstly, the consistency of the measurement system is validated. The influences of incident angle and sensitive element size on the temporal pressure profiles are also analyzed. Secondly, the peak pressures, decay constant of the temporal pressure waveforms obtained by the two kinds of probes are thoroughly compared. The dependence of peak pressures on propagation distances are studied. Thirdly, the feasibility of peak pressure evaluation using the TOF method based on pressure probes is discussed. Finally, a new pressure waveform model is proposed. Based on the Fourier transform and energy conservation law, the pressure waveform obtained by the PCB138 probe is reconstructed using this model to approach to the real pressure signal.

## 2. Measurement System

### 2.1. Experimental Setup

**Figure 1:**
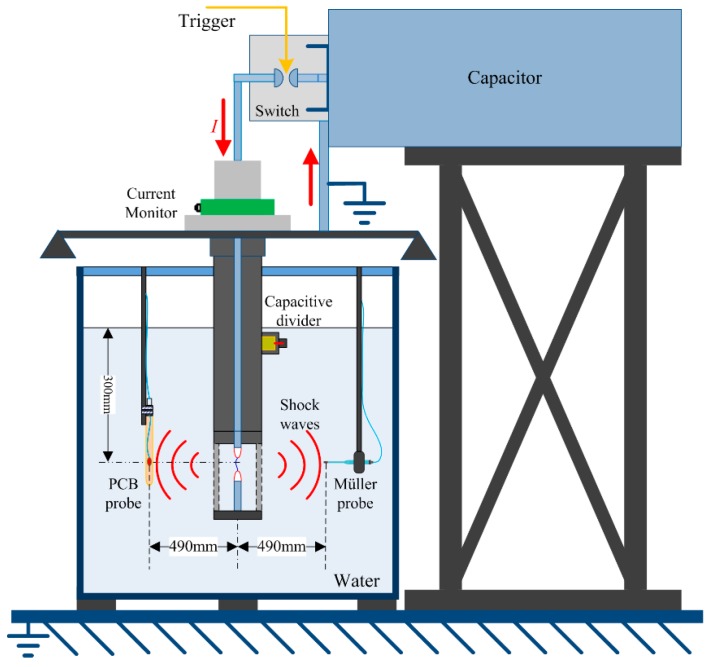
Schematic diagram of the SW generator based on UEWE.

We applied underwater electrical wire explosion (UEWE) technology to generate shock waves. The experimental setup is shown in Figure 1 and has been presented in our previous work [23,24,25,26]. A pulsed capacitor of ~6 μF with the maximum charging voltage of 50 kV was connected to a discharge load through a coaxial triggered switch. The discharge load was a pair of electrodes connected with a thin exploding wire. As the electrical energy deposits into the wire, the fast expansion of volume caused by both phase explosion and plasma discharge channel expansion could generate strong SWs.

**Figure 2:**
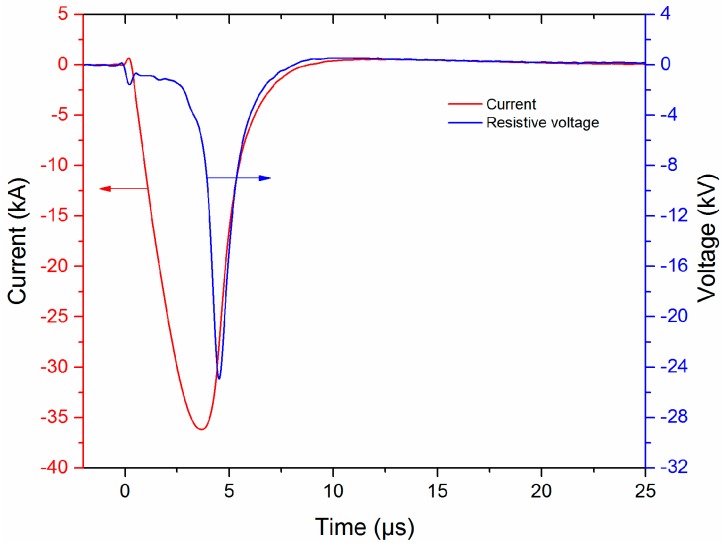
Typical waveforms of current and resistive voltage.

The typical waveforms of the current and resistive voltage waveforms are shown in Figure 2. When the capacitor is charged to the voltage of 19.8 kV and the discharge load is a 50 mm length, 280 μm diameter copper wire, a pulsed current with the amplitude of 36.2 kA and a rise time of ~3.5 μs could be obtained. The resistive voltage can be obtained by subtracting the inductive voltage from the measured voltage, and specific calculating method can be found in references [23,27]. The recording digital oscilloscope adopts the discharge current as the trigger source, not the switch’s trigger signal, so the uncertainty of trigger process will not affect the measurement of the SW arrival times.

The SW probes adopted in this paper were two PCB138A11 pressure probes and two Müller-plate needle hydrophones. Important parameters for two kinds of pressure probes are listed in Table 1. The PCB138 series underwater blast sensors, which are produced by PCB Piezotronics, Inc. (Depew, New York, NY, USA), have been widely used and commonly accepted around the world because of their easy installation, high repeatability and good performance. While the Müller-plate probes can give a faster SW front than the PCB138 probes [13,28], but are relatively harder to install in practical application and can only measure direct-arrival SW signal due to its single directivity. Besides, the Müller-plate probe (Dr. Müller Instruments, Oberursel, Germany) is a kind of differential probe and often fail to return to zero, which makes energy calculations impossible with this kind of probe [29]. The PCB138 probes are calibrated under static pressure of up to 10 MPa, while the Müller-plate probes are calibrated in a water shock tube using a shock wave with a known peak pressure value. So far, both PCB138 and Müller-plate probes have been widely used and taken as standards for the relative pressure measurements of SWs.

**Figure 3:**
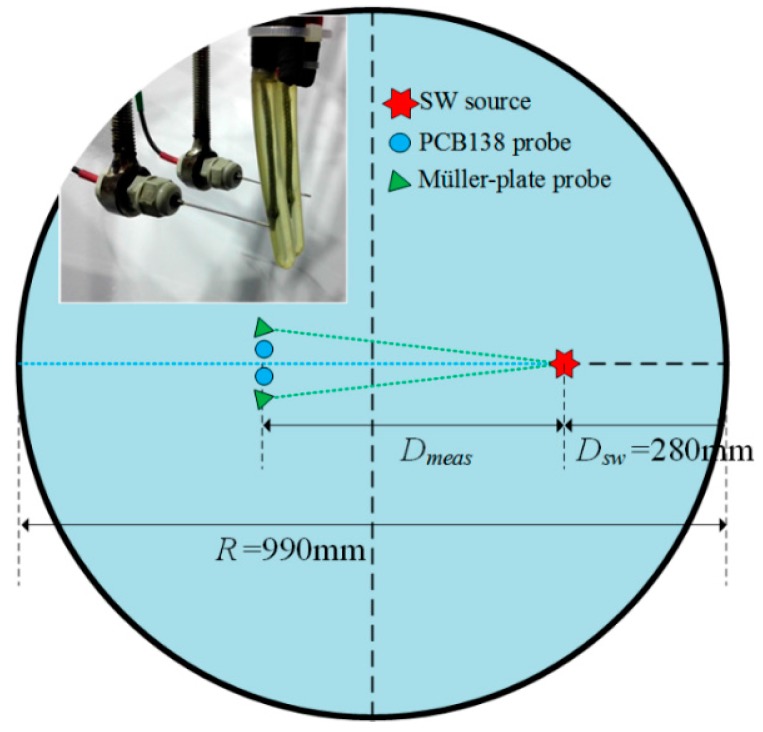
Pressure probes arrangements in the water tank.

The experiments were carried out in a cylindrical water tank with the size of Φ990 mm×1200 mm and the arrangements of pressure probes were shown in Figure 3. The SW source was placed at the distance of 280 mm (*D*_sw_) from the internal wall of the tank. The pressure probes could be moved at the distance range of 200–560 mm (*D*_meas_) from the SW source. At these distances, the SW could be regarded as a plane wave. The distances between four pressure probes and the SW source were almost the same and precisely measured before the experiment. At each distance, at least five repeated tests were produced with the same discharging parameters. The SW source and the pressure probes were placed at the deep enough position (water depth of 350 mm) to avoid the possible influence of the “bulk cavitation phenomena [30]” effects.

### 2.2. Measurement System Consistency

In our study, the UEWE technique is adopted to generate underwater SWs. In each test, a new metal wire has to be reinstalled to serve as a discharge load, so the position of the SW source may change slightly. Between different tests, the parameters of the metal wire and the charging voltage of the capacitor cannot be completely the same, so the discharge process and the generated SWs may also differ from each other slightly. In this paper, we aimed to study the performances of pressure probes, so a good measurement reliability is definitely required to ensure the measuring repeatability. The possible influence factors on the measurement consistency will be experimentally studied and discussed below.

**Figure 4:**
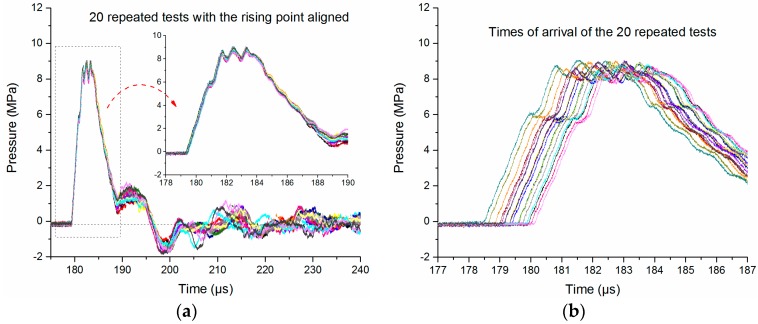

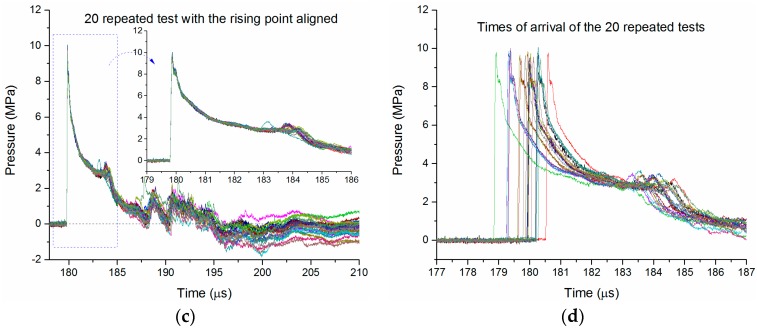
20 repeated temporal pressure waveforms at the distance of 260 mm from discharge channel. (**a**) Pressure waveforms obtained by the PCB138 probe with the SW arrival times aligned; (**b**) distribution of the SW arrival times obtained by the PCB138 probe; (**c**) pressure waveforms obtained by the Müller-plate probe with the SW arrival times aligned; (**d**) distribution of the SW arrival times obtained by the Müller-plate probe.

First, the SW source must be very stable during the whole experiment, so that the SWs generated in different tests will have a good comparability and repeatability. Keeping the experimental conditions (circuit structure, charging voltage, exploding wire parameters and the measure system) unchanged, 20 repeated tests are produced. The resulting typical current and voltage waveforms are shown in Figure 2, and the corresponding temporal pressure waveforms obtained by both the PCB138 probe and the Müller-plate probe at the distance of 260 mm from discharge channel are shown in Figure 4. The aligned SW pressure waveforms are shown in Figure 4a,c. It is evident that both the waveforms obtained by both PCB138 probes and Müller-plate probe are almost overlapped at the early stage (for example 10 μs), and then begin to diverge from each other at longer times. Especially for the Müller-plate probe, the deviations in the wave tail are larger and unpredictable, and what is even worse, many measured signals fail to return to zero, so any energy or impulse calculations based on the Müller-plate probe will be inaccurate.

In Figure 4a,c, the arrival times have been aligned to examine the repeatability of all the SW waveforms. In fact, the SW arrival times of different signals are discrepant, as shown in Figure 4b,d. The peak pressure values and arrival times of the 20 pressure waveforms are obtained and statistically analyzed, and then their mean value, standard deviation and coefficient of variance are calculated, as given in Table 2.

The coefficients of variance of the peak pressures obtained by both pressure probes are less than 2%, which proves that the SW source is stable and reliable. Besides, we think the existing errors of peak pressures may be explained by the instability of the SW source.

In addition to the instability of the SW source, the distance error between the SW source and the pressure probe may also affect the consistency of the measurement system. As shown in Table 2, the coefficients of variance of arrival times obtained by two kinds of probes are less than 0.3%, and the standard deviations are less than 0.5 μs. Assuming that the speed of weak SWs is close to the SW speed in uncompressed water (~1470 m/s), the corresponding distance error is less than 0.735 mm, which indicates that the SW propagation distance error between different tests is very small. The structure of the experimental setup is very stable, and the arrangement of the pressure probes is very precise.

In the above experiments, the positions of the pressure probes remained unchanged. When the position of the probe changes, an extra distance error may be introduced, so its influence on the peak pressure also has to be analyzed. Assume the dependence of peak pressure on distance meets the formula given in Equation (1) [13] in the rather far field, and then we can obtain the relative error of *P*_peak_ with Equation (2):

(1) $$P_{peak}\propto r^{-0.5}$$

(2) $$\frac{\Delta P_{peak}}{P_{peak}}=\frac{{\left(r+\Delta r\right)}^{-0.5}-r^{-0.5}}{r^{-0.5}}$$

where *r* is the propagation distance between the SW source and the pressure probe, Δ*r* is the distance error, and Δ*P*_peak_ is the peak pressure error. Considering the PCB138 probe has a relatively large size (9.6 mm in diameter), the installation error may be within ±5 mm. When *r*_0_ = 260 mm and ±Δ*r* = 5 mm, the relative error of the *P*_peak_ is −0.95/+0.98%. When *r*_0_ = 560 mm and ±Δ*r* = 5 mm, the relative error of the *P*_peak_ is −0.44/+0.45%. It is obvious that a distance error of less than 5 mm will not produce a peak pressure error larger than 1% in the distance range of 260–560 mm. That is, the probe does not have to be installed precisely for measuring the peak pressure.

To summarize, the consistencies of the two kinds of pressure probes are both excellent, and the whole measurement system is very stable and reliable.

### 2.3. Influence of Incident Angle and Sensitive Element Size

**Figure 5:**
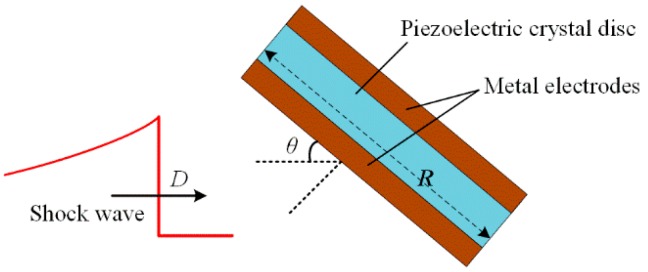
Sensitive element of a piezoelectric probe.

Although the consistency of the measurement system has been well verified, the measured waveforms may still change conspicuously after the reinstallation of pressure probes, and the waveforms obtained by two pressure probes of the same type may have obvious distinctions. This may be explained by the difference of the incidence angles. The incidence angle *θ* can be defined as the angle between the SW propagation direction and the tangential direction of the thin piezoelectric crystal disc, as shown in Figure 5. The signal obtained by the metal electrodes is proportional to the integration of the piezoelectric charges. Considering the SWs’ propagation speed is finite, one can draw the conclusion that the pressure field establishment at the sensitive element is a transient process, so the rising time of obtained signal is determined by the time required for the SW to cover the sensitive element totally, which is sensitive to the incidence angle *θ*.

**Figure 6:**
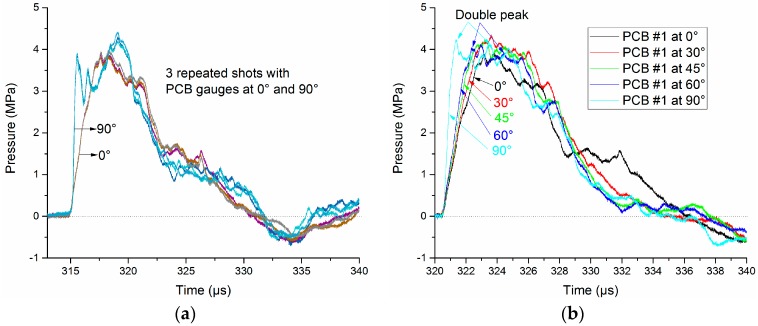
Pressure signals obtained by the PCB138 probe with different incident angles at the distance of 460 mm (**a**) Consistency of the obtained pressure waveforms; (**b**) rising edge of pressure signal with different incident angles.

The measured data have confirmed our analysis. Figure 6 shows pressure signals obtained by the same PCB138 probe with different incidence angles. The PCB138 series pressure probes are installed vertically, and the incidence angle *θ* can vary from 0° to 90°. In order to reduce the random errors, three repeated tests for each incidence angle *θ* were produced, and the obtained data (*θ* = 0° and 90°, typically) show a good consistency, as shown in Figure 6a. The rising edge is affected heavily by the increase of *θ*. Along with the increase of *θ*, the pressure field establishment time will decrease accordingly, as shown in Figure 6b. Besides, the waveform distortion becomes more severe with the increase of *θ*, and a “double peak” phenomenon even appears when *θ* = 60° and 90°. This may be caused by the scattering effect.

In practical applications, the PCB138 probes should be placed with the thin crystal disc facing the incoming SW edge-on (*θ* = 0°) to avoid any possible interference or scattering effects, while the incidence angle for the Müller-plate probe is always 90° because it shall be installed facing the SW source. However, the sensitive element diameter of a Müller-plate probe is less than 0.5 mm, so the scattering effect is not remarkable.

The size of the sensitive element determines the sensitivity and the spatial resolution, and it also affects the upper-frequency response to a great extent [15]. An oversized measuring device will disturb the medium, while an undersized sensitive element has a small piezoelectric constant and the signal to noise ratio (SNR) of signal is too low, so choosing a suitable size is very necessary for free field pressure measurements. Aside from the probe size and incidence angle, media acoustic impedance matching, the signal processing circuit, and other factors may also affect the measurement accuracy.

## 3. Analysis of Signals Obtained by PCB138 and Müller-Plate Probes

**Figure 7:**
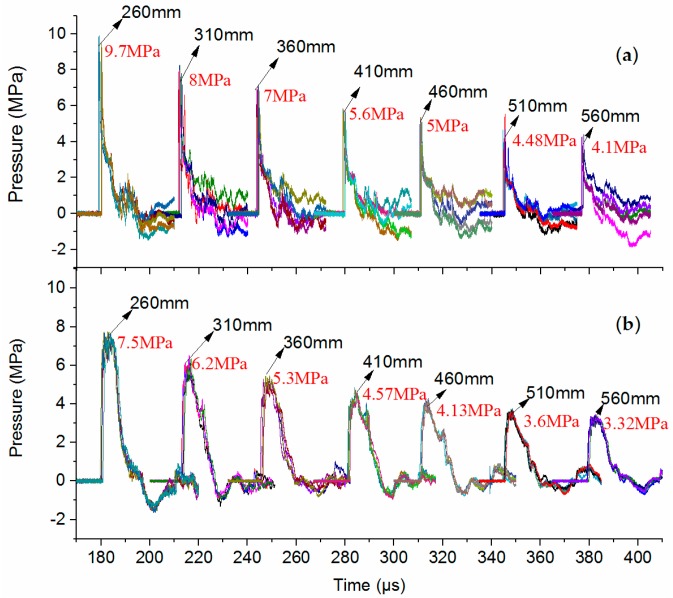
Temporal waveforms obtained by pressure probes *vs.* distances. (**a**) Müller-plate probe; (**b**) PCB138 probe.

Based on the excellent and stable measurement system described above, the performance of the PCB138 and Müller-plate probes will be analyzed and discussed comprehensively in this section. In order to study the SW attenuation characteristics, seven observation points were set at distances of 260, 310, 360, 410, 460, 510 and 560 mm apart from the discharge channel. The temporal pressure waveforms obtained by both PCB138 and Müller-plate probes are shown in Figure 7. At each distance, the five waveforms obtained by the PCB138 probes in repeated tests are almost overlapped, while for the Müller-plate probes, after a certain time delay, along with the appearance of some unknown oscillations, the waveforms obviously deviate from each other, and the measurement repeatability becomes bad. At present, we have no definite explanation for this deviation, but obviously, this kind of pressure probe is not a practical alternative for impulse and energy calculations.

An ideal free field temporal SW pressure signal *P*(*t*) at a certain distance from the blast source can be described by:

(3) $$P\left(t\right)=\varepsilon \left(t-t_{0}\right)P_{m}e^{-\frac{\left(t-t_{0}\right)}{\tau }}$$

where *P*_m_ is the peak pressure, *τ* is the decay time constant, *ε*(*t* − *t*_0_) is the step function, and *t*_0_ is the SW arrival time. Within the time duration of one *τ*, Equation (3) can provide a fair approximation for SWs to the time of one *τ*, and then the SW decay rate slows down in longer times [15]. The time constants *τ* can be defined as the time required for the pressure to decay from *P*_m_ to the pressure value *P_τ_* = *P*_m_/e.

Peak pressure and decay time constant are most important parameters for an SW, and we will provide further analysis of these terms below.

### 3.1. Analysis of the Peak Pressure vs. SW Propagating Distance

**Figure 8:**
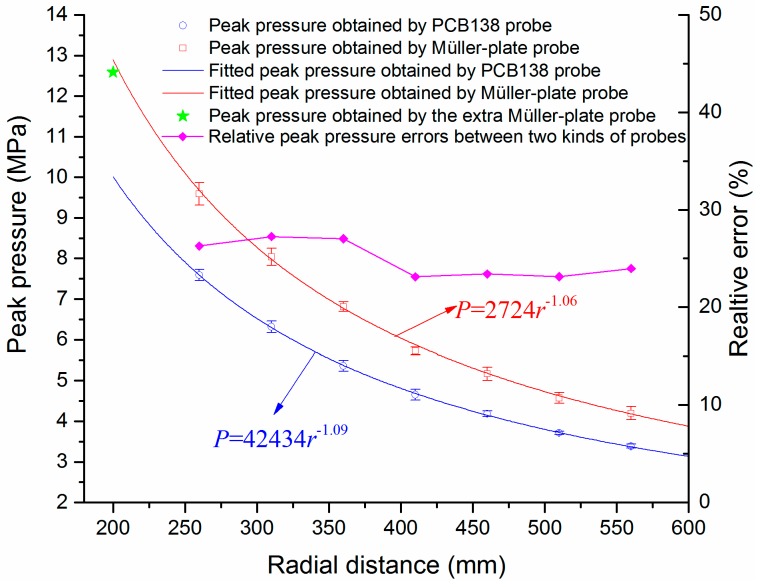
Dependence of the SW peak pressures and fitted values on the distance from the discharge channel.

First, the five peak pressure values of both probes at each distance given in Figure 7 are extracted and averaged, and the mean peak pressures are shown in Figure 8. Then the dependence of the SW peak pressures on the propagating distance is fitted with Equation (4):

(4) $$P_{peak}=P_{0}r^{-\alpha }$$

where *r* is the distance between pressure probe and the discharge channel, and *P*_0_ and *α* are unknown constants to be determined. When the coefficient of determination (*R*-square) is the closest to 1, the two well-fitted results, which are *P*_peak_ = 42434*r*^−1.09^ and *P*_peak_ = 2724*r*^−1.06^, can be obtained separately, as shown in Figure 8. In order to verify the accuracy of the fitting results, we placed an extra observation point at the radical distance of 200 mm. The measured peak pressure by the Müller-plate probe is ~12.59 Mpa and is marked with a green star in Figure 8. It is obvious that this measured peak pressure value has a very good accordance with the predicted value given by the fitting curve, so the fitting results are fairly accurate.

Theoretically, the SWs will finally evolve into spherical acoustic waves in the far field, and the dependence of peak pressures on propagation distances in the far field will follow the function *P*_peak_ = *P*_0_*r*^−1^. In this paper, the distances range between the observation points and the SW source are 260–560 mm. In this distance range, the SW has not been fully attenuated, so the decay rate of peak pressures should be faster than that in the far field, *i.e.*, the corresponding decay constant *α* should be larger than 1. The fitting results illustrated in Figure 8 can prove our analysis. Besides, the decay constants *α* (1.06 and 1.09) for the Müller-plate probe and the PCB138 probe are quite close to each other, so the decay characteristics of SW peak pressure obtained by two kinds of probes are similar. Furthermore, the relative errors of peak pressures between the Müller-plate probe and the PCB138 probe at different radial distances vary in a very small range of 23%–27%, as shown in Figure 8. The approximately constant errors indicate that the two kinds of pressure probes both have good uniformities and linearities. In fact, it is very hard to obtain the absolutely accurate peak pressure value of SWs. Measuring the relative pressure precisely is more meaningful in practical application. Due to the good stability and linearity, both the PCB138 probe and the Müller-plate probe can be regarded as standards for relative peak pressure measurement.

### 3.2. Analysis of the Pulse Width and Decay Time Constant

**Figure 9:**
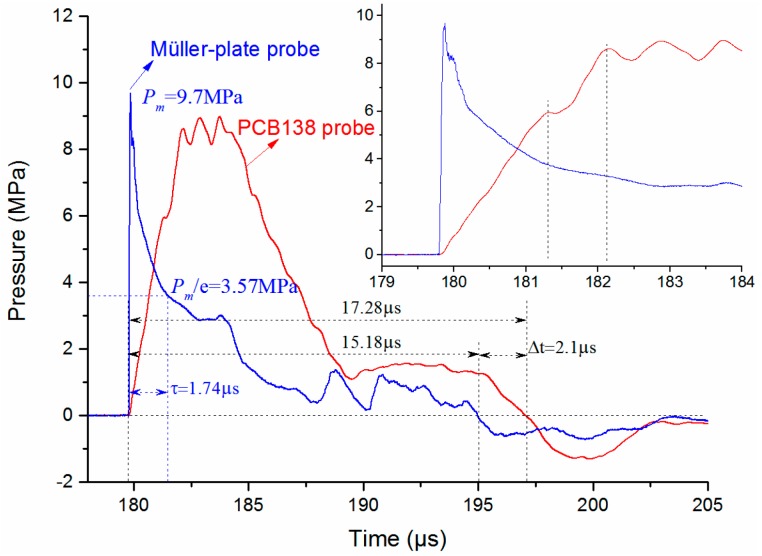
Typical time-dependent pressure waveforms obtained with both pressure probes at the distance of 260 mm.

Decay constant, as another important parameter for an SW signal, should also be well analyzed. In order to reduce the random error, 20 repeated SW signals in Figure 4a,c are averaged, and the pressure waveforms are shown in Figure 9. The pressure signal obtained by the Müller-plate probes has a much smaller rise time (~50 ns) than the PCB138 probes (more than 1 μs), which is much closer to the ideal SW waveform. We define the pulse width as the time duration of the positive pulse. The pulse width of the signal obtained by the PCB138 probe is little larger than by the Müller-plate probe. This may be caused by the relatively bigger size (~Φ3.2 mm × 1 mm) of the sensitive element of the PCB138 probe and the 0° incidence angle. The relatively larger size will consume extra time for the SW front to cover the whole tourmaline disc, and we suppose this will extend the propagation time duration on the sensitive element. The extra time can be calculated by Equation (5):

(5) Δt=(Rcosθ)/D

where *R* is the diameter of the piezoelectric crystal disc, *D* is the SW speed in the medium and *θ* is the incident angle, as shown in Figure 5. Considering the SW speed in silicone oil is ~1350 m/s, *R* = 3.2 mm and *θ* = 0°, one can calculate the extra time Δ*t* = 2.37 μs, which is very close to the measured Δ*t* (2.1 μs), as shown in Figure 7. That is to say, after subtracting the extra SW propagation time, the pulse widths obtained by the two kinds of probes are almost the same. This indicates that our analysis is correct. We may reach the conclusion that both kinds of probes are accurate enough for measuring the SW pulse width.

**Figure 10:**
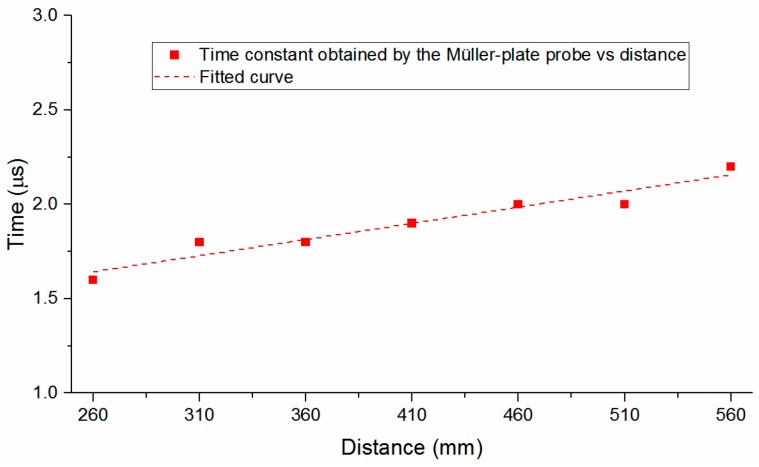
Decay constants obtained by the Müller-plate probe *vs.* distance.

Further, through analyzing the SW signal obtained by the Müller-plate probe, one can find that the waveform coincides with the ideal SW signal given in Equation (5) in one *τ*. Then the decay time constant τ can be calculated. The decay constants of the five SW signals measured at each distance shown in Figure 7a are calculated and averaged, and the dependence of the decay constant on the SW propagating distance is illustrated in Figure 10. At the distance of 260 mm, the time constant has the minimum value, and increase overall along with the increase of the propagation distance, which accords with the SW attenuation characteristics and verifies the high accuracy of the probe somehow.

Although the PCB138 probe and the Müller-plate probe both have good reliability in measuring SW peak pressures and pulse widths or decay constants, their measured waveforms are heavily distorted. In order to obtain more accurate pressure waveforms, the measured signals may need to be reconstructed.

## 4. SW Pressure Evaluation and Reconstruction

Because of the bandwidth limitations of the pressure probes, the measured signals are not accurate enough to represent the real SW. The TOF method is a theoretically accurate way to estimate the peak pressure of SW. If this method can be easily performed in practical applications, it will be very helpful and attractive. Except for the peak pressure, the waveform measurement is also important. The directly obtained signals by the pressure probes are usually heavily distorted, so a waveform reconstruction method is required to get the real signals.

### 4.1. Peak Pressure Evaluation with the TOF Method

#### 4.1.1. SW Pressure Measurement Principle Based on the TOF Method

The TOF method is based on the EOS of water and the Rankine-Hugoniot Equation. If the undisturbed water is static, the SW front can be described by Equation (6):

(6) $$(\begin{array}{l} \rho (D-u)=\rho_{0}D\\ \rho_{0}Du=P-P_{0}\\ E-E_{0}=\frac{1}{2}\left(P+P_{0}\right)\left(\frac{1}{\rho_{0}}-\frac{1}{\rho }\right) \end{array}$$

where *P*, *ρ*, *u*, *D* and *E* are pressure, density, particle velocity, SW velocity and internal energy behind the SW front, respectively; *P*_0_, *ρ*_0_, and *E*_0_ are density and internal energy of undisturbed water, respectively. When the pressure is less than 2.5 GPa, the EOS of water is shown in Equation (7) [19]:

(7) $$P=A\left[{\left(\frac{\rho }{\rho_{0}}\right)}^{n}-1\right]+P_{0}$$

where A = 3.045 × 108 Pa and n = 7.15 is the adiabatic constant of water [31]. Substituting Equation (7) into Equation (6), one can obtain SW velocity behind SW front in water, as described by Equation (8) [22]:

(8) $$D^{2}=\frac{\rho }{\rho_{0}}\frac{P-P_{0}}{\rho -\rho_{0}}=\frac{P-P_{0}}{\rho_{0}\sqrt[{n}]{\frac{P-P_{0}}{A}+1}\cdot \left(\sqrt[{n}]{\frac{P-P_{0}}{A}+1}-1\right)}$$

**Figure 11:**
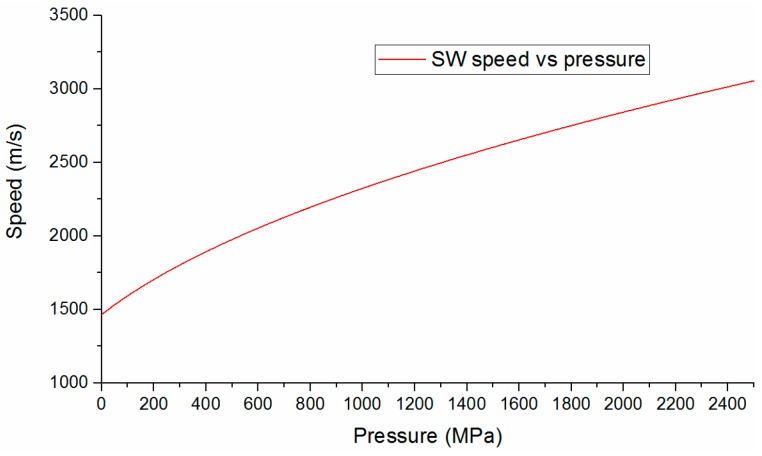
Dependence SW speed behind shock front on pressure.

The relations of SW speed and pressure is shown in Figure 11. When the SW speed is known, the pressure value can be easily obtained.

#### 4.1.2. Feasibility of Peak Pressure Evaluation with the TOF Method in Practical Application

As given by Equation (8) the pressure is a function of SW speed, but the expression is very complex. In order to easily analyze the theoretical error of the TOF method, we give an approximate polynomial, as described by Equation (9):

(9) $$P=511D^{2}-785839D+47386945$$

When the SW speed is less than 1800 m/s, the approximation is rather accurate. In experiments, the SW speed *D* at a certain distance from the SW source is calculated with *D* = *s_p_*/*t_p_*, in which, *s_p_* is the distance between two pressure probes and *t_p_* is the time lag of the pressure signals obtained by two pressure probes. *D* is the average speed between the two probes. If *s*_p_ is too large, the error of *D* will be rather large due to the nonlinear propagation of the SW.

The error of peak pressure Δ*P* can be calculated by Equation (10). Usually, using the TOF method to measure the peak pressure value are not easily achieved, even in labs, because the error propagation is prominent. The precise measurements of the distances between the pressure probes are often difficult to accomplish for two reasons. First, the pressure probe has a finite size, instead of being an ideal point. Second, the installation and distance measurement of pressure probes in the underwear environment is inconvenient, so some installation errors become inevitable, and this will lead to large errors in calculating the peak pressure. For example, we assume *s*_p_ = 50 mm, *D* ≈ 1600 m/s, *t*_p_ = *s*_p_/*D* = 31.25 μs, the distance error caused by installation Δ*s*_p_ ≈ 1.6 mm, and the time error Δ*t*_p_ = 0, which indicates, except for the distance error Δ*s*_p_ caused by installation, all parameters are precisely measured. Then the absolute error of pressure Δ*P* will be ~43.5 Mpa according to Equation (10):

(10) $$\Delta P=\left|511\frac{2s_{p}}{t_{p}^{2}}-785839\frac{1}{t_{p}}\right|\left(\Delta s_{p}+\frac{s_{p}}{t_{p}}\Delta t_{p}\right)$$

**Figure 12:**
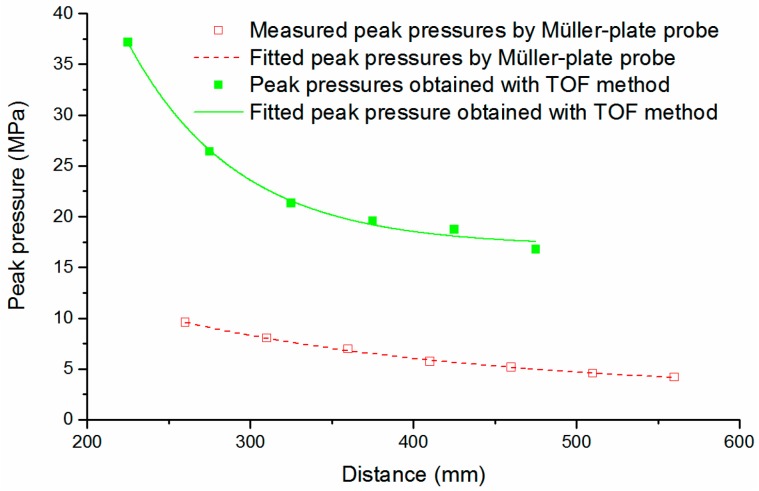
SW peak pressures obtained by the TOF method.

That is, a small installation error (Δ*s*_p_) of probes will lead to a rather large peak pressure error. Figure 12 shows our experimental and calculated results. We adopted two Müller-plate probes to measure the arrival times of the SW, because their sensitive elements are rather small (<0.5 mm in diameter), and the introduced distance error are smaller than with the PCB138 probes. The Müller-plate probes are carefully placed at the distances of 200, 250, 300, 350, 400, 450 and 500 mm from the discharge channel. Results show that the calculated peak pressures with the TOF method are overall larger than the values directly obtained with the Müller-plate probe, and this support our error analysis above.

The error cannot be easily controlled unless the experimental setup and the measurement system are specially designed to ensure that the distances are precise, and the pressure probes are small enough. Using streak shadow imaging or multi-frame camera imaging is a good choice to get precise propagation distances at a certain time delay [32,33], but the shock speed *D* can only be diagnosed in a rather small distance range due to the limited field of view. In a word, we think that the TOF method cannot be easily realized to measure the peak pressure using the finite-sized pressure probes in practical applications, unless a precise timing device and accurate distance measurement system are available.

### 4.2. Reconstruction of the Real Pressure Waveform with the Energy Conservation Method

According to the analysis in Section 3, the PCB138 probe has better consistency, but it has a lower frequency band of no more than 1 MHz, while the Müller-plate probe has a rather high-frequency response (11 MHz), but it behaves stably just in the time of one *τ*, and is unpredictable and discrepant in longer times. Besides, the PCB138 probe can be easily installed, while the Müller-plate probe has to be installed facing the SW source. Furthermore, the PCB138 probe has been widely used in practical applications, so reconstructing the SW signals based on PCB138 probes is more meaningful than the Müller-plate probes. The signal of a Müller-plate probe has a more precise wavefront and wave tail within a certain time duration, so the Müller-plate probe signal is also adopted here to verify the wave reconstruction results.

#### 4.2.1. Waveform Reconstruction Criteria

In order to restore a more precise SW profile, we developed a new wave reconstruction method based on the method described in [21]. The time-domain characteristics (including peak pressure, pulse width, *etc.*) and the frequency domain characteristics (energy distribution, *etc.*) of the restored signal should be much closer to the real signal than the measured signal.

The energy distributions of an SW signal in both time domain and frequency domain are finite. Referring to [21], we adopt two assumptions as our waveform reconstruction criteria. First, we suppose that, at a certain frequency *f*_0_ in the lower frequency band, the magnitude frequency response of the reconstructed signal *P*_rec_ should theoretically be equal to that of the measured signal *P*_m_. Another criterion is based on the energy conservation law. According to Parseval’s theorem, the signal energy in the time domain is equal to that in the frequency domain. If two signals have similar energy characteristics in the time domain, their energy distribution in the frequency domain should also be similar. The frequency response of the PCB138 probe in the low-frequency range (for example 1–10 kHz) is flat and unity, we can suppose the measured signal in this frequency range is real and reliable, so the energy in the frequency domain of the restored signal *E*_rec_ should be equal to that of the measured signal *E*_m_ in this specific frequency range. The two waveform reconstruction criteria can be described by Equation (11):

(11) $$\left\{ \begin{array}{l} P_{rec}\equiv \int_{-\infty }^{\infty }p_{rec}\left(t\right)\exp^{-j2\pi t•f_{0}}dt=P_{m}\equiv \int_{-\infty }^{\infty }p_{m}\left(t\right)\exp^{-j2\pi t•f_{0}}dt\\ E_{rec}\equiv {\int_{f_{l}}^{f_{h}}\frac{\left|P_{rec}\left(f\right)\right|}{\rho_{0}c_{0}}}^{2}df=E_{m}\equiv {\int_{f_{l}}^{f_{h}}\frac{\left|P_{m}\left(f\right)\right|}{\rho_{0}c_{0}}}^{2}df \end{array}\right.$$

where *p*_m_ and *p*_rec_ are the pressure values of measured signal and the reconstructed signal. *f*_l_ and *f*_h_ are the lower and upper limit frequencies of the integral. *P*_m_ and *P*_rec_ are the frequency response of *p*_m_ and *p*_rec_.

Theoretically, according to Equation (11), *P*_rec_ should be strictly equal to *P*_m_, and *E*_rec_ should be strictly equal to *E*_m_. However, in the calculation, some errors are inevitable. For example, the sample length cannot be infinite, and some frequency spectrum leakage is also unavoidable. The SW measurement with the pressure probes cannot be exactly accurate, so satisfactory reconstruction results may not be achieved only with Equation (11). In order to obtain more accurate waveform reconstruction results, we propose the reconstruction criteria based on the minimum errors, which require that both the magnitude error *ErrorP* and the relative error *ErrorP* be rather small. The two new criteria are given by Equation (12):

(12) $$\left\{ \begin{array}{l} ErrorP\left(f_{0}\right)=\left[P_{rec}\left(f_{0}\right)-P_{m}\left(f_{0}\right)\right]/P_{m}\left(f_{0}\right)\\ ErrorE=\left[\left(E_{rec}-E_{m}\right)/E_{m}\right] \end{array}\right.$$

Because the lower cut-off frequency of the PCB138 probe is 2.5 Hz, taking *f*_0_ = 0 Hz as the calculation condition is not reasonable. In this paper, we take *f*_0_ = 1 kHz, *f*_l_ = 1 kHz and *f*_h_ = 10 kHz as the calculation conditions. The threshold values for both *ErrorP* and *ErrorE* are both set to 1.5% to get the right reconstructed signal parameters.

#### 4.2.2. Three Kinds of SW Waveform Models

From Figure 9, we suppose that the real SW pressure signal should have a sharp shock front, but the signal given by the PCB138 probe is distorted, so we propose three types of waveforms with a faster rise time and a much slower wave tail to reconstruct the measured pressure signal, which are a triangular decay signal, an exponential decay signal and a multi-exponential decay signal, respectively, as given in Equations (13)–(15):

(13) $$p_{tri}=\left\{ \begin{array}{ll} 0, & t<t_{0}\text{ and }t>t_{0}+\Delta T\\ -\left(\frac{p_{peak}}{\Delta T}\right)t+p_{peak}\left(1+\frac{t_{0}}{\Delta T}\right), & t_{0}\leq t\leq t_{0}+\Delta T \end{array}\right.$$

(14) $$p_{\exp}=\left\{ \begin{array}{ll} 0, & t\leq t_{0}\\ p_{peak}\exp\left(-\frac{t-t_{0}}{\tau }\right), & t\geq t_{0} \end{array}\right.$$

(15) $$p_{comp}=\left\{ \begin{array}{ll} 0, & t\leq t_{0}\\ p_{peak}\exp\left(-\frac{t-t_{0}}{\tau }\right), & t_{0}\leq t\leq t_{0}+\tau \\ p_{peak}B^{\left(-\frac{t-t_{0}}{\tau }\right)}\cdot \frac{p_{peak}\exp\left(-\frac{\tau -t_{0}}{\tau }\right)}{p_{peak}B^{\left(-\frac{\tau -t_{0}}{\tau }\right)}}, & t\geq t_{0}+\tau \end{array}\right.$$

where *P_peak_* is the peak pressure value of a signal, Δ*t* is pulse width of the triangular signal, *t*_0_ is the arrival time of the SW, *τ* is the time constant of an exponential decay signal and *B* is a constant. We set *B* = 2 in this paper.

#### 4.2.3. Waveform Reconstruction Process

The process of restoring the real SW signal can be described as follows:

(1) A series of peak pressure *P*_peak_, pulse width Δ*t* and time constant *τ* were assumed in a rather large value range, so SW waveforms with different parameters could be obtained with Equations (13)–(15).
(2) FFT calculations were performed, and the magnitude error *ErrorP* and the relative error *ErrorP* were calculated with Equation (12).
(3) The proper peak pressure *P*_peak_, pulse width Δ*t* and time constant *τ* could be picked up with the pre-set threshold values of both *ErrorP* and *ErrorE,* and the real SW signal can be obtained.

#### 4.2.4. Typical SW Reconstruction Results

**Figure 13:**
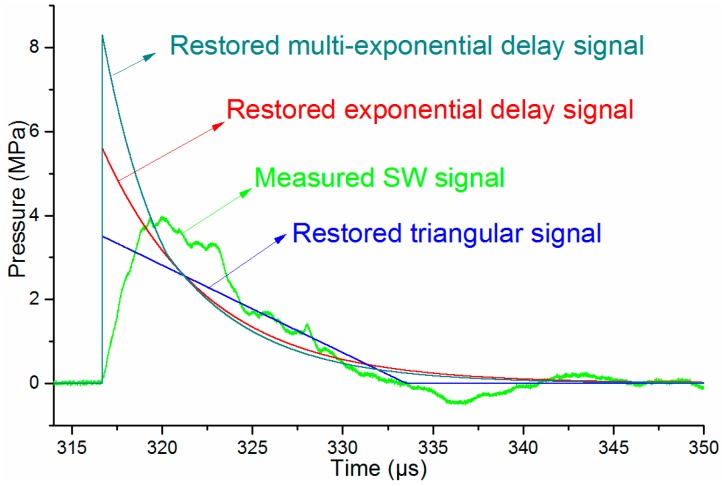
Reconstructed pressure signals based on three types of waveforms.

A typical pressure waveform measured at the distance of 460 mm from the discharge channel with the PCB138 probe at the incidence angle of 0° is adopted as the signal to be reconstructed. Then, based on the three types of waveforms and the two waveform reconstruction criteria above, three restored signals are shown in Figure 13. The magnitude errors *ErrorP* at the frequency *f*_0_ for a triangular signal, exponential decay signal, and multi-exponential decay signal are 0.68%, 1.21% and 1.17%, respectively, and the energy errors *ErrorE* for the three signals are 0.46%, 1.23% and 0.97% separately.

As shown in Figure 13, the peak pressure of the restored triangular signal is slightly smaller than that of the measured signal. The peak pressures of both the exponential decay signal and the multi-exponential decay signal are larger than the measured one. The peak pressure value of the multi-exponential decay signal is nearly twice the measured peak pressure. Considering the SW characteristics, it is reasonable that the reconstructed waveforms based on the exponential decay signal and the multi-exponential decay signal models are more accurate than the directly measured signal.

#### 4.2.5. Verification of the Reconstruction Results

**Figure 14:**
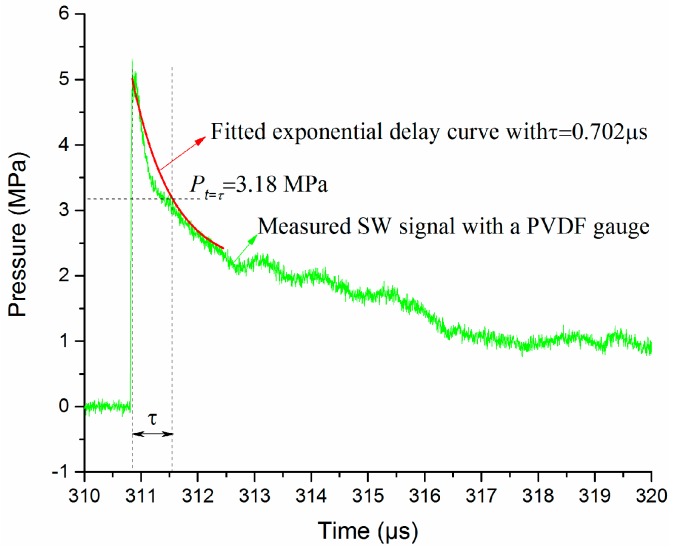
Peak pressure evaluation with the Müller-plate probe signal.

In order to examine the accuracy of the wave reconstruction algorithm, we propose a peak pressure evaluation method based on the pressure waveform obtained by the Müller-plate probe. Because the Müller-plate probe has a rise time of less than 50 ns and a high upper cut-off frequency of 11 MHz, we suppose its wavefront and wave tail within one *τ* are relatively accurate. The wave tail should agree with the exponential decay formula. With the help of a curve fitting calculation, the time constant *τ* can be obtained. Then, the pressure value *P*_t = *τ*_ when time equals to one *τ* can be obtained. Finally, a peak pressure can be calculated by *P*_m_ = *P*_t = *τ*_ × e. As shown in Figure 14, the pressure signal is measured by a Müller-plate pressure probe at the same distance as the PCB138 probe shown in Figure 13. At time *τ*, the fitted pressure *P*_t = *τ*_ = 3.18 Mpa, and the calculated *P*_m_ = 8.64 Mpa. This calculated peak pressure is quite close to the value given by the multi-exponential decay signal shown in Figure 13. This validates the feasibility and validity of the waveform reconstruction algorithm.

#### 4.2.6. Discussion

Through the previous analysis, the finite size of the sensitive element is an important reason to limit the high-frequency response of a probe and reduce the measurement accuracy of the peak pressure. The real SW front has a nanosecond rise time. If the size of the sensitive element is comparable with the wavelength of the SW, the wavefront will be stretched, the peak pressure value will be reduced. Besides, the PCB138 probe is calibrated with static pressure. Its sensitivity is credible in static or quasi-static pressure conditions, but under high-frequency pressure conditions, the nominal sensitivity may be lower than the real one. Thesetwo reasons may explain why the peak pressures restored with exponential and multi-exponential decay signals are larger than the measured peak pressure.

Besides, the measured pressure signal is sampled discretely during a finite length of time. The frequency resolution is determined by the sample length. The measurement in a finite size water tank determines that the sample length cannot be very long, because the obtained signal will be superposed by the reflected waves caused by the inner wall or water surface. Besides, along with the fast decay of the SW signal, the pressure values approach zero, and then the SNR becomes very low, so a reasonable waveform interception is important. Furthermore, the discrete Fourier transform will inevitably lead to spectrum leakage. All these influencing factors may affect the FFT results, and thus, decrease the accuracy of the waveform reconstruction algorithm.

## 5. Conclusions

The performances of two commercial piezoelectric pressure probes in measuring the SWs generated by UEWEs are analyzed, and the measured signal is reconstructed based on the proposed SW signal model. Some conclusions can be drawn: (1) The experimental setup and the measurement system are verified to be reliable and accurate; (2) The PCB138 probe has better repeatability, while for the Müller-plate probe, the repeatability can only be assured during the early stage (for example, in less than one *τ*), and then the measured wave tails become unreliable and may fail to return to zero; (3) The incidence angle and the finite size of the sensitive element will obviously affect the rise time of the PCB138 probe. In order to measure the SW pressure field more precisely, smaller sensitive element and suitable incident angle may be acquired; (4) Both the PCB138 and the Müller-plate probes can be used to measure the relative SW pressure value because of their good uniformities and linearities. The pulse width obtained by the PCB138 probe is slightly larger than by the Müller-plate probes, which can be explained by the much larger sensitive element size of the PCB138 probe than the Müller-plate probe; (5) The TOF method is a theoretically accurate way to measure SW peak pressure, but it may not be easily achieved in practical application, because the arrival times and the distances cannot be measured precisely; (6) The reconstructed waveforms based on the multi-exponential decay model are closer to the ideal SW waveforms than the measured signals. The reconstruction accuracy has been verified by the signals obtained by the Müller-plate probe. The waveform reconstruction method is reasonable and reliable.

## Figures and Tables

**Table 1:** Parameters of the PCB138 probe and the Müller-plate needle hydrophone.

Parameters	PCB138A11	Müller-Plate Needle Hydrophone
Sensitive element type	Tourmaline	PVDF
Sensitive element size	~Φ3.2 mm × 1 mm (measured)	<Φ0.5 mm
Sensitivity uncertainty	±15%	-
Rise time	<1.5 μs	<50 ns
Bandwidth	2.5–1 MHz	0.3–11 MHz
Cable length	20 m	2 m

**Table 2:** Statistical analysis of peak pressures and arrival times.

Parameters	PCB138 Probe		Müller-Plate Probe	
	Peak Pressures	Arrival Times	Peak Pressures	Arrival Times
Mean value	8.87 MPa	179.91 μs	9.70 MPa	179.80 μs
Standard deviation	0.136 MPa	0.460 μs	0.196 MPa	0.427 μs
Coefficient of variance	1.5%	0.26%	2.0%	0.24%
